# Evaluation of Dermatological and Neurological Aspects of the Relationship between Rosacea and Headaches

**DOI:** 10.3390/diagnostics14010023

**Published:** 2023-12-22

**Authors:** Merve Alizada, Turgut Sahin, Ozden Sener, Pelin Kocyigit

**Affiliations:** 1 Department of Dermatology, Mamak State Hospital, 06230 Ankara, Turkey; 2 Department of Neurology, Cankırı State Hospital, 18100 Çankırı, Turkey; turgutsahin@ankara.edu.tr; 3 Department of Neurology, Ankara University Faculty of Medicine, 06100 Ankara, Turkey; osener@medicine.ankara.edu.tr; 4 Department of Dermatology, Ankara University Faculty of Medicine, 06100 Ankara, Turkey; kocyigit@medicine.ankara.edu.tr

**Keywords:** rosacea, primary headaches, migraine, neurogenic inflammation, vascular dysregulation

## Abstract

This study aimed to investigate the relationship between rosacea and headaches, focusing on different subtypes, as well as the associated clinical features and triggering factors. In this prospective study, 300 patients diagnosed with rosacea and 320 control subjects without rosacea or any connected mast cell activation illness were included. Patients with rosacea were assessed by a dermatologist according to the 2019 updated rosacea classification (ROSCO panel). Accordingly, patients were classified based on their predominant rosacea subtype as follows: erythematotelangiectatic (ETR), papulopustular (PPR), or phymatous (RhR). Patients experiencing headaches were assessed using the International Headache Classification. Headaches were categorized as migraine, tension-type headaches (TTHs), secondary types (STHs), and cluster-type headaches (CTHs). The ratio of headache was 30.3% in the rosacea group, which did not show a significant difference compared to the control group (30.3% vs. 25.0%, *p* = 0.138). In 81.3% of rosacea patients with headaches, headache onset occurred after the diagnosis of rosacea. The rate of patients with headaches was higher in the ETR group compared to the PPR and RhR groups (35.2% vs. 16.2% vs. 23.1%, *p* = 0.007, respectively). In terms of headache subtypes, the rates of patients with migraine and STHs were higher in the ETR group compared to the PPR and RhR groups, while the rate of patients with TTHs was higher in the RhR group. A positive correlation was found between rosacea severity and migraine severity (r = 0.284, *p* < 0.05). Among the triggering factors for rosacea, only sunlight was found to be associated with headaches. Lower age, female gender, and moderate to severe rosacea severity were identified as independent factors increasing the likelihood of headaches. A significant portion of rosacea patients experience headaches. Particularly, different subtypes of rosacea may be associated with various types of headaches. This study, highlighting the connection between migraine and ETR, is a pioneering work that demonstrates common pathogenic mechanisms and potential triggers.

## 1. Introduction

Rosacea is a chronic skin disorder characterized by flushing, erythema, telangiectasia, papulopustular lesions, and ocular manifestations, typically exhibiting a fluctuating course with episodes of exacerbation and remission. The prevalence of rosacea is reported to be between 2–22% [1]. Epidemiological studies have demonstrated an association between rosacea and headaches, with a particular emphasis on migraines [2,3]. This is linked to a common pathophysiology involving neurovascular dysregulation and inflammation [4,5,6].

Both rosacea and migraine are commonly triggered by factors such as physical and mental stress, specific foods and drinks, exposure to ultraviolet light, as well as extreme temperatures [7,8,9]. Additionally, these conditions have been linked to anxiety and depression, significantly impacting the quality of life [9]. On the other hand, headaches include primary types such as tension-type headaches (TTHs), migraines, and cluster-type headaches (CTHs), as well as secondary types (STHs) that arise from pathological conditions [10]. Although recent studies have underscored a possible association between migraines and rosacea, a thorough investigation into the relationship between rosacea and various other types of headaches has not yet been conducted.

We hypothesized that there might be a significant relationship between rosacea and headaches across various subtypes. Therefore, this study aimed to investigate the relationship between rosacea and headaches, focusing on different subtypes, as well as the associated clinical features and triggering factors. 

## 2. Materials and Methods

Following the principles set forth in the Declaration of Helsinki, this prospective study was conducted at the Ankara University Dermatology and Neurology Department from January 2020 to June 2021. The study received approval from the local ethics committee (Approval Date: February 2020, Decision No. I2-77-20). All participants provided their written informed consent.

### 2.1. Study Population

The study included 300 patients diagnosed with rosacea and 320 control subjects without rosacea or any connected mast cell activation illness. Patients under 18 years of age, those not providing written informed consent, those with any connected mast cell activation illness such as accompanying allergic disease, atopic dermatitis, allergic eczematous dermatitis, or urticaria, those with inflammatory, auto-immune, or photosensitive diseases, or those with diseases causing fibrosis in the skin like scleroderma were excluded from the study.

### 2.2. Study Protocol

The assessment process involved a comprehensive evaluation conducted by both a dermatologist for skin conditions and a neurologist for neurological aspects. A standardized questionnaire was employed to identify typical triggers in both rosacea and headache subgroups. A detailed medical history, current comorbidities, demographics, and lifestyle factors such as smoking habits, alcohol use, caffeine intake, and sun exposure were collected through a structured questionnaire administered by an interviewer (Supplementary Materials File S1).

**Figure 1:**
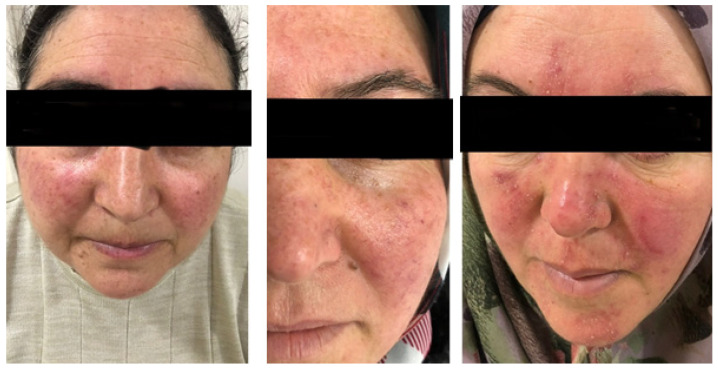
Patients with the predominant rosacea subtypes of ETR and PPR. On the left, a 55-year-old patient with prominent burning, stinging, and itching symptoms and a moderate severity of ETR for 35 years. In the middle, a 46-year-old patient with moderate severity ETR for 28 years, without burning, stinging, or itching symptoms. On the right, a 42-year-old patient with prominent erythema, burning, stinging, and itching symptoms, and a moderate severity of PPR for 27 years.

Patients with rosacea were assessed by a dermatologist according to the 2019 updated rosacea classification (ROSCO panel). Accordingly, patients were classified based on their predominant rosacea subtype as follows: erythematotelangiectatic (ETR), papulopustular (PPR) (Figure 1), or phymatous (RhR) [11]. They were photographed, and the severity of rosacea in each patient was determined using the National Rosacea Society Rosacea Clinical Scorecard (Supplementary Materials File S1) [12]. All occurrences of burning, stinging, itching, and dryness experienced by patients with rosacea were documented.

Neurological assessments in patients were carried out by a neurologist, and the findings were documented along with the patients’ medical histories. Patients experiencing headaches were assessed using the International Headache Classification [13]. Headaches were categorized as migraine, TTHs, STHs, or CTHs. For measuring headache severity, the Migraine Disability Assessment (MIDAS) Questionnaire was recorded in migraine patients (Supplementary Materials File S1) [14], while the VAS score during attacks was noted in patients with other types of headaches [15].

### 2.3. Statistical Analysis

Statistical analyses of all data were conducted using IBM SPSS Statistics for Windows, version 20.0 (IBM Corp., Armonk, NY, USA). The normal distribution of numerical data was determined using the Kolmogorov–Smirnov test. Data meeting normality criteria were expressed as mean ± standard deviation, while those not meeting the criteria were presented as median (min–max). Categorical variables were shown as numbers and percentages. The assessment of differences between two groups in numerical variables was carried out using either the Student’s T-test or the Mann–Whitney U test, contingent upon the normality of the data. For analyses involving three or more groups, the Anova test (post hoc: Bonferroni test) or Kruskal–Wallis H test (post hoc: Dunn’s test) were employed. For the comparison of categorical variables, the Chi-Square test and Fisher’s Exact test were utilized. The relationship between numerical variables was investigated using Pearson and Spearman correlation analyses. Independent factors affecting headaches in rosacea patients were evaluated using multivariable logistic regression analysis with the backward method. A *p*-value ≤ 0.05 was considered statistically significant.

## 3. Results

**Figure 2:**
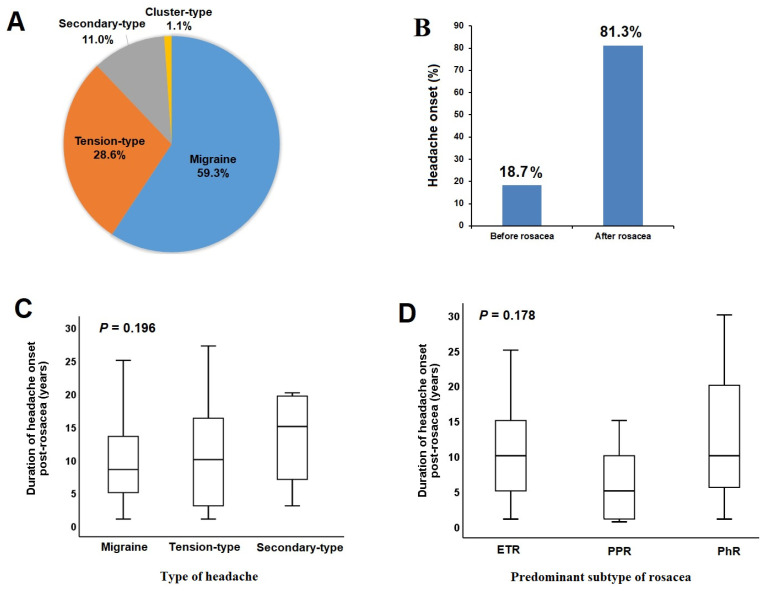
Distribution of headache types (**A**) and onset timing (**B**) in rosacea patients with headaches. Comparison of headache onset duration post-rosacea diagnosis across headache types (**C**) or rosacea predominant subtypes (**D**).

In patients with rosacea, the mean age was 45.8 ± 12.5 years (range = 18–73 years), with a predominant female majority. The median duration of the disease was 16 years (range = 1–50 years), and 40.7% of the patients reported a family history. Among the rosacea patients, 73% (*n* = 219) had the predominant ETR subtype, followed by 23% (*n* = 68) with predominant PPR subtype, and 4% (*n* = 13) with predominant RhR subtype. In 60% of the patients diagnosed with ETR (*n* = 132), only ETR symptoms were observed, while the remaining patients exhibited concurrent symptoms such as papules, pustules, and rhinophymatous changes. The ratio of headaches was 30.3% in the rosacea group, which did not show a significant difference compared to the control group (30.3% vs. 25.0%, *p* = 0.138). In rosacea patients who experienced headaches, 25.3% (*n* = 23) reported mild severity, 37.4% (*n* = 34) had headaches of moderate severity, and 37.4% (*n* = 34) faced severe headaches. In these patients, migraines were most frequently observed (Figure 2A). It was also noted that among the rosacea patients with migraines, 61% had migraines without episodic aura, 37% had migraines with episodic aura, and 2% experienced menstrual migraines. The ratio of patients with migraine-type headaches was higher in the rosacea group compared to the control group (18.0% vs. 9.0%, *p* = 0.011) (Table 1). 

In 81.3% of rosacea patients with headaches, headache onset occurred after the diagnosis of rosacea (Figure 2B), with a median headache onset duration of 10 years (range = 1–30 years). The median onset duration of headaches following the diagnosis of rosacea did not show significant differences across headache types (Figure 2C) or rosacea subtypes (Figure 2D). A positive correlation was found between rosacea severity and migraine severity (r = 0.284) (*p* < 0.05).

Demographic and clinical findings by predominant subtypes of rosacea are presented in Supplementary Table S1. The rate of patients with headaches was higher in the predominant ETR subtype group compared to the predominant PPR subtype and predominant RhR subtype groups (35.2% vs. 16.2% vs. 23.1%, *p* = 0.007, respectively). In terms of headache subtypes, the rates of patients with migraine and STHs were higher in the predominant ETR subtype group compared to the predominant PPR subtype and predominant RhR subtype groups, while the rate of patients with TTHs was higher in the predominant PhR subtype group. Other headache characteristics, such as severity and triggering factors, did not show significant differences among predominant subtypes of rosacea (Supplementary Table S2).

In rosacea patients with headaches, the mean age was lower (42.6 ± 10.2 vs. 47.2 ± 13.2, *p* = 0.003), and the ratios of female and patients with peptic ulcer were higher compared to those without headaches. Other demographic characteristics of rosacea patients did not show significant differences between those with and without headaches. The prevalence of burning, stinging, and itching symptoms was higher in rosacea patients with headaches compared to those without (51.6% vs. 30.6%; *p* = 0.001). Other rosacea symptoms did not show significant differences between the groups. The ratio of patients with moderate and severe rosacea was higher among those with headaches than those without. Among the triggering factors for rosacea, only sunlight was found to be associated with headaches (Table 2).

The mean age was higher in patients with STHs compared to those with other types of headaches. The distribution of comorbidities did not show significant differences based on the type of headache. The rates of burning, stinging, itching, and facial edematous symptoms were higher in patients with migraine-type headaches compared to those with other types of headaches. The rate of patients with severe rosacea was higher in the migraine group. No significant relationship was found between the triggering factors of rosacea and the types of headaches (Supplementary Table S3).

The ratio of patients reporting stress as a headache trigger was higher in the TTH group, while those reporting coffee, menstruation, and cheese as triggers were more prevalent in the migraine group. In the STH group, a higher ratio of patients identified hypertension as a headache trigger (Supplementary Table S4).

**Figure 3:**
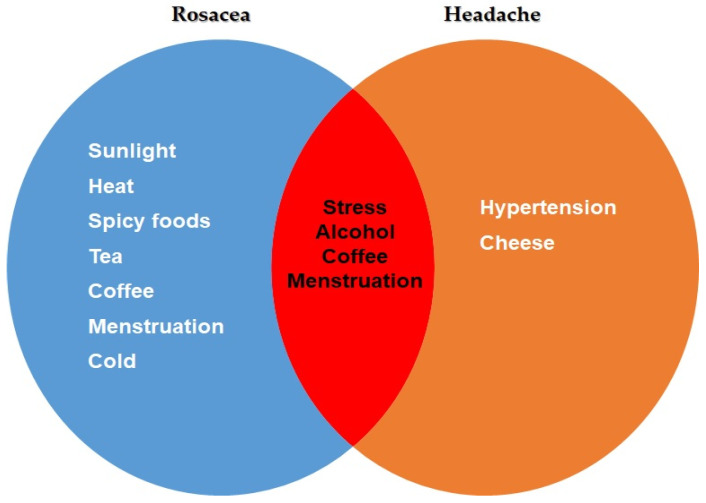
Common trigger factors for headache and rosacea patients.

Stress, alcohol, coffee, and menstruation were identified as common triggering factors for both rosacea and headaches (Figure 3) (Supplementary Table S5).

In a multivariable regression model incorporating factors associated with headaches in rosacea patients, lower age, female gender, and moderate to severe rosacea severity were identified as independent factors increasing the likelihood of headaches (Table 3).

## 4. Discussion

This study stands as a pioneering effort, offering a comprehensive analysis of the relationship between rosacea and headaches based on disease type and triggering factors. Although previous studies have investigated the relationship between rosacea and migraines, this connection has not been explored in the context of predominant subtypes of rosacea and headache subtypes. In rosacea patients, particularly in the predominant ETR subtype, migraine-type headaches were observed more frequently. Furthermore, it was found that headaches frequently emerge subsequent to the onset of rosacea. Lower age, male gender, and rosacea severity had an independent association with headaches.

Consistent with epidemiological studies, which report a headache prevalence of 12% to 54% [3,5,6], patients with rosacea had a higher frequency of headaches compared to the control group. In a meta-analysis study conducted by Stovner et al., which investigated the global prevalence of headaches, it was shown that TTH is the most prevalent type of headache in the general population [16]. Although TTH is the most prevalent primary headache type in the overall population, it is noteworthy that migraine was the most common primary headache in the rosacea group [5,17]. Although these findings indicate a lower likelihood of TTHs in rosacea,, the underlying pathogenesis of TTH is not yet clearly understood [18]. Therefore, this topic deserves further research. On the other hand, the frequency of CTHs were reported to be around 0.5% in both the rosacea and control groups [6]. In a study conducted using national data registers in Denmark, the initial prevalence of migraine was reported as 7.3% in the general population and 12.1% in patients with rosacea. Additionally, it has been shown that the likelihood of developing migraine in patients with rosacea is 1.3-fold higher than in the general population and being over 50 years of age and of female gender are independently associated [3]. In a United Kingdom-based national study that used a reverse approach to investigate the risk of rosacea development in patients with migraine, it was reported that female migraine patients had a 1.2-fold increased risk of rosacea, while this association was not observed in males. Additionally, it was reported that in women, the risk of rosacea increases with age [6]. In the current study, although the duration of rosacea was similar in patients with and without headaches, the mean age was lower. However, in the migraine patients, approximately 96% were female. Moreover, consistent with the studies reported above, female gender had an independent association with the likelihood of developing headaches.

The relationship between rosacea and headaches might be linked to multiple underlying mechanisms. Inflammatory processes, a fundamental aspect of rosacea, could influence neural pathways and contribute to headache development. Previous studies have reported a positive correlation between the duration and severity of rosacea and its inflammatory response [19,20]. In the current study, it was found that a majority of patients with headaches developed their headaches after being diagnosed with rosacea. A shared vascular change between rosacea and certain headache types, along with neurogenic modulation, might play a role [21,22]. The variability in headache types among different rosacea predominant subtypes further suggests a complex interplay, where specific inflammatory and pathophysiological features of each subtype differentially impact neurological outcomes. Neurogenic inflammation and vasodilation are apparent in both the aura and headache phases of a migraine [23,24]. The trigeminal ganglion and its nerve fibers are connected to the meninges and dural vessels, and their stimulation leads to the release of vasoactive neuropeptides, including substance *p* and calcitonin gene-related peptide. This release culminates in neurogenic inflammation, vasodilation, and migraine pain [25]. Furthermore, the release of neuropeptides can lead to migraine attacks and cause rosacea-like erythema and flushing [26,27]. This potential mechanism may explain the high rates of migraine in rosacea patients with a predominant ETR subtype. Additionally, mast cells, which are part of the innate immune system, connect inflammatory vascular and neurogenic arrangements in rosacea, creating the microvascular unit of the skin. Mast cell counts have been found to be raised in all predominant subtypes of rosacea, especially PPR, even from the beginning [28]. Mast cells contribute to the inflammatory response in rosacea by producing LL-37 and matrix metalloproteinases (MMPs)-9 [7]. In rosacea, it has been reported that the Th17 pathway, which is thought to influence LL-37, is activated and there is an increase in interleukin (IL)-17 [29,30]. In rosacea patients, increased IL-17 levels are associated with inflammation, angiogenesis, and the stimulation of LL-37 and MMP-9 expression [31,32]. On the other hand, factors of both innate and adaptive immunity, including mast cells which are closely linked to dural vessels and nerve pathways that trigger pain sensation, play a significant role in migraines. They stimulate vasodilation, inflammation, and heightened vascular permeability [24,33]. In patients with migraines, an increase in IL-17 and MMP activity has been detected, independent of aura [34,35]. These potential mechanisms, particularly the varying levels of mast cell release in different types of rosacea, may lead to variations in headache types.

The trigger factors of rosacea may play a significant role in the development of headaches following rosacea. Among the physical factors reported to trigger rosacea symptoms, exposure to sunlight, stress, and hot weather are the most frequently mentioned [36]. Bright light, including sunlight, serves both as a trigger and an exacerbating factor for migraine attacks, making individuals with migraines susceptible and vulnerable to the effects of sunlight [37,38]. In this current study, the association of sunlight, a known trigger factor for rosacea, with headaches can be explained. While stress was the most common trigger for headaches in rosacea patients, it was also the second most frequent trigger for rosacea itself. This is consistent with studies suggesting that stress may be a common triggering factor for both rosacea and headaches [39,40]. On the other hand, the low prevalence of TTHs in patients with rosacea does not necessarily mean that these patients experience less stress compared to healthy controls. In the current study, 92.3% of rosacea patients with TTHs reported stress as a triggering factor for their headaches. A meta-analysis study showed that 19.6% of rosacea patients suffer from depression and 15.6% from anxiety. It also revealed that the likelihood of exhibiting symptoms of depression and anxiety is at least twice as high in rosacea patients compared to healthy controls [41]. Transient receptor potential vanilloid (TRPV) receptors, located in various anatomical sites such as dermal nerve endings, the trigeminal ganglion, and scalp arteries, respond to fluctuations in stress and temperature. Rosacea patients exhibit an increased density of receptors and a lower threshold for stimulation, indicating the potential for therapeutic applications in both rosacea and migraines [42,43]. Certain studies propose that the PAR2 receptors, existing alongside the TRPV receptors, may interconnect the neurogenic and inflammatory pathways in both afflictions [43,44]. The precise role of neurogenic inflammation in migraine pathogenesis remains a topic of debate, although it is thought to influence chronicity and pain perception [42,43]. Remarkably, it was observed that the majority of rosacea patients suffering from headaches, particularly migraines, predominantly presented with the ETR subtype, characterized by symptoms such as burning, stinging, and itching. These patients may be more sensitive to migraine-triggering factors such as sunlight [45].

This study had several significant limitations. There were age differences both in the control group and among the rosacea subtypes. The age discrepancy may have negatively influenced the prevalence of migraine in rosacea patients [21]. Inflammatory parameters were not assessed in the study. Additionally, comorbid conditions were not matched between groups. Evaluating these factors could have played a role in shedding light on the inflammation mechanism between headache and rosacea [46].

## 5. Conclusions

A significant portion of rosacea patients experience headaches. Particularly, different predominant subtypes of rosacea may be associated with various types of headaches. This study, highlighting the connection between migraine and the predominant ETR subtype, is a pioneering work that demonstrates common pathogenic mechanisms and potential triggers. The predominant ETR subtype, categorized as a subtype of rosacea with diverse clinical features and insufficient treatment outcomes, may denote a distinctive patient cohort, which demonstrates classic rosacea symptoms, but stems from different pathogenic mechanisms. These findings could lead to fresh therapeutic strategies.

## Figures and Tables

**Table 1:** Characteristic features of the study population.

Variables	Control Group *n* = 320	Rosacea Group *n* = 300	*p*-Value
Age, years	38.5 ± 16.0	45.8 ± 12.5	0.001 *
Gender, *n* (%)			
Male	208 (65.0)	214 (71.3)	0.091
Female	112 (35.0)	86 (28.7)	
BMI, kg/m^2^	26.2 ± 4.2	26.5 ± 4.6	0.396
Smoking, *n* (%)			
Never	230 (71.8)	223 (74.3)	0.582
Current	62 (19.4)	55 (18.4)	
Former	28 (8.8)	22 (7.3)	
Alcohol use, *n* (%)	9 (2.8)	6 (2.0)	0.517
History of family, *n* (%)	-	122 (40.7)	-
Duration of disease, years	-	16 (1–50)	-
Predominant subtype of rosacea, *n* (%)			
ETR	-	219 (73.0)	-
PPR	-	68 (23.0)	
RhR	-	13 (4.0)	
Headache, *n* (%)	80 (25.0)	91 (30.3)	0.138
Migraine	30 (9.0)	54 (18.0)	0.011 *
TTH	35 (11.0)	26 (8.7)	
STH	15 (5.0)	10 (3.3)	
CTH	-	1 (0.3)	

Data are mean ± standard deviation or median (min–max), or number (%). * *p*-value < 0.05 shows statistical significance. BMI, body mass index; CTH, cluster-type headache; ETR, erythematotelangiectatic-type rosacea; PPR, papulopustular-type rosacea; RhR, phymatous-type rosacea; STH, secondary-type headache; TTH, tension-type headache.

**Table 2:** Demographic and clinical findings in rosacea patients based on the presence of headaches.

Variables	Headache		*p*-Value
	No *n* = 209	Yes *n* = 91	
Age, years	47.2 ± 13.2	42.6 ± 10.2	0.003 *
Gender, *n* (%)			
Male	76 (36.4)	10 (11.0)	<0.001 *
Female	133 (63.6)	81 (89.0)	
BMI, kg/m^2^	27.2 ± 4.5	26.7 ± 4.7	0.363
Fitzpatrick skin type, *n* (%)			
Type II	56 (26.8)	22 (24.2)	0.670
Type III–IV	153 (73.2)	69 (75.8)	
Smoking, *n* (%)			
Never	160 (76.6)	63 (69.2)	0.354
Current	34 (16.3)	21 (23.1)	
Former	15 (7.2)	7 (7.7)	
Alcohol use, *n* (%)	4 (1.9)	2 (2.2)	0.999
History of family, *n* (%)	78 (37.3)	44 (48.4)	0.074
Comorbidities, *n* (%)	115 (55.0)	59 (64.8)	0.113
Peptic ulcer	104 (49.8)	58 (63.7)	0.026 *
Hypertension	57 (27.3)	19 (20.9)	0.312
Diabetes mellitus	34 (16.3)	19 (20.9)	0.329
Thyroid disease	23 (11.0)	9 (9.9)	0.842
Depression	14 (6.7)	2 (2.2)	0.162
Anxiety	6 (2.9)	1 (1.1)	0.679
Inflammatory bowel disease	3 (1.4)	-	0.556
Predominant subtypes of rosacea, *n* (%)			
ETR	142 (67.9)	77 (84.6)	0.007 *
PPR	57 (27.3)	11 (12.1)	
RFR	10 (4.8)	3 (3.3)	
Accompanying symptoms, *n* (%)			
Flushing	30 (14.4)	14 (15.6)	0.859
Burning, stinging, itching	64 (30.6)	47 (51.6)	0.001 *
Dryness	93 (44.5)	51 (56.0)	0.066
Facial edematous	12 (5.7)	11 (12.1)	0.096
Granulomatous changes	-	1 (1.1)	0.668
Rosacea severity, *n* (%)			
Mild	82 (39.2)	13 (14.3)	<0.001 *
Moderate	86 (41.1)	51 (56.0)	
Severe	41 (19.6)	27 (29.7)	
Duration of disease, years	15 (1–50)	18 (3–47)	0.757
Positive demodex test, *n* (%)	87 (41.6)	33 (36.3)	0.383
History of treatment, *n* (%)	44 (21.1)	27 (29.7)	0.139
Topical	42 (20.1)	23 (25.3)	0.361
Systemic	22 (10.5)	10 (11.0)	0.999
Triggering factors of rosacea, *n* (%)			
Sunlight	122 (58.4)	67 (73.6)	0.013 *
Stress	58 (27.8)	35 (38.5)	0.078
Heat	57 (27.3)	22 (24.2)	0.669
Spicy foods	43 (20.6)	23 (25.3)	0.367
Alcohol	1 (0.5)	1 (1.1)	0.515
Tea	9 (4.3)	5 (5.5)	0.767
Coffee	6 (2.9)	6 (6.6)	0.233
Menstruation	1 (0.5)	1 (1.1)	0.999
Cold	2 (1.0)	3 (3.3)	0.335

Data are mean ± standard deviation or median (min–max), or number (%). * *p*-value < 0.05 shows statistical significance. BMI, body mass index; ETR, erythematotelangiectatic-type rosacea; PPR, papulopustular-type rosacea; RhR, phymatous-type rosacea.

**Table 3:** Independent predictors of increased headache risk in rosacea patients.

Variables	Univariable Regression			VIF	Multivariable Regression		
	OR	95% CI	*p*		OR	95% CI	*p*
Age	0.97	0.95–0.99	0.004 *	1.23	0.95	0.93–0.98	<0.001 *
Gender				1.09			
Male	ref				ref		
Female	4.63	2.27–9.46	<0.001 *		3.59	1.68–7.70	0.001 *
Peptic ulcer	1.77	1.07–2.94	0.026 *	1.23	-	-	-
Predominant subtypes of rosacea				1.08			
ETR	2.6	1.37–4.92	0.003 *		3.07	1.52–6.19	0.002 *
Others	ref						
Burning, stinging, itching	2.42	1.46–4.01	0.001 *	1.26	-	-	-
Rosacea severity				1.27			
Mild	ref				ref		
Moderate	3.74	1.90–7.38	<0.001 *		4.92	2.35–10.32	<0.001 *
Severe	4.15	1.94–8.89	<0.001 *		5.00	2.17–11.50	<0.001 *
Triggering factors of rosacea							
Sunlight	1.99	1.16–3.42	0.013 *	1.04	-	-	-
					Nagelkerke R^2^ = 0.318		

* *p*-value < 0.05 shows statistical significance. CI, confidence interval, ETR, erythematotelangiectatic-type rosacea; OR, odds ratio; VIF, variance inflation factor.

## Data Availability

The data that support the findings of this study are available on request from the corresponding author. The data are not publicly available due to privacy.

## Supplementary Materials

The following supporting information can be downloaded at: https://www.mdpi.com/article/10.3390/diagnostics14010023/s1, File S1. Rosacea—Headache Patient Evaluation Form; Table S1. Demographic and clinical findings according to predominant subtypes of rosacea; Table S2. Headache findings according to predominant subtypes of rosacea; Table S3. Demographic and clinical findings according to headache type in rosacea patients; Table S4. Other findings according to headache type in rosacea patients; Table S5. Common trigger factors for headache and rosacea patients. References [47,48] are cited in the supplementary materials.
