# Supplementary material for: Evaluation of Dermatological and Neurological Aspects of the Relationship between Rosacea and Headaches
Source: Diagnostics (Basel). 2023 Dec 22;14(1):23. doi: 10.3390/diagnostics14010023 (PMC10795644; doi:10.3390/diagnostics14010023)
Supplement: Supplementary file 1 [file diagnostics-14-00023-s001.zip › Supplementary materials File S1.pdf]

Rosacea - Headache Patient Evaluation Form

Patient consent form:

Patient name / age / gender / photographs:

Fitzpatrick skin type:

Dominant rosacea subtype (Erythematelengiectatic rosacea- papulopustular rosacea- rhinophimatous rosacea- ocular rosacea) (1)

Rosacea disease severity (according to the Rosecea Clinical Scorecard - mild - moderate - severe) (2)*

Presence of telangiectasia:

Presence of flushing / persistent erythema:

Presence of papulopustular lesions:

Presence of phimatous changes:

Ocular manifestations (burning, stinging, redness of the eye):

Other symptoms accompanying rosacea (burning, stinging, itching, granulomatous changes, oedema in the central part of the face):

How many years have you had rosacea? When (at what age) did the clinical signs of rosacea first start?

Is there a family history of rosacea?

Are there triggers that increase rosacea?

What are the triggers for rosacea? (Uv Radiation, alcohol, smoking, spicy foods, temperature increase, emotional stress, tea-coffee consumption, exercise, menstruation, medications, cold, cheese-chocolate consumption)

Does the patient have headache?

What is the headache subtype (Primary headache (tension-type headache, migraine, cluster headache), Secondary headache)**(3)

At what age did the headache start?

When did the headache start in relation to the onset of rosacea (before - after)?

Is there a family history of headache?

Is there a triggering factor for headache?

What are the triggering factors for headache? (Uv Radiation, alcohol, smoking, spicy foods, temperature increase, emotional stress, tea-coffee consumption, exercise, menstruation, medications, cold, cheese-chocolate consumption, hypertension)

Are there common triggers for rosacea with headache? What are the common triggers?

Are there aggravation/exacerbation of rosacea symptoms during headache periods?

Does the patient have migraine?

How old did migraine start?

When did migraine start according to the onset of rosacea (before - after)?

Is there a family history of migraine?

Is there a family history of both rosacea and migraine?

What is the migraine subtype (episodic migraine without aura, episadic migraine with aura, hemiplegic migraine, retinal migraine, vestibular migraine, menstrual migraine, ocular migraine)?

What is the severity of migraine (MIDAS stages 1-2-3-4) (4)?

Are there triggers that increase migraine?

What are the triggers that increase migraine? (Uv Radiation, alcohol, smoking, spicy foods, temperature increase, emotional stress, tea-coffee consumption, exercise, menstruation, medications, cold, cheese-chocolate consumption)

Increased symptoms of rosacea during migraine attacks - is there an exacerbation?

Are there common triggers for migraine and rosacea? If so, what are the common triggers?

Is there a relationship between migraine and rosacea subtypes?

Is there a relationship between clinical findings accompanying rosacea and frequency and severity of migraine?

What are the clinical characteristics of patients with exacerbation-increase in rosacea symptoms during migraine attacks?

NATIONAL ROSACEA SOCIETY

Rosacea Clinical Scorecard

Patient Name ____________________________ Date: _____________________

Primary Features

Flushing (transient erythema)	☐ Absent	☐ Mild	☐ Moderate	☐ Severe
Nontransient erythema	☐ Absent	☐ Mild	☐ Moderate	☐ Severe
Papules and pustules	☐ Absent	☐ Mild	☐ Moderate	☐ Severe
Telangiectasia	☐ Absent	☐ Mild	☐ Moderate	☐ Severe

Secondary Features

Burning or stinging	☐ Absent	☐ Mild	☐ Moderate	☐ Severe
Plaques	☐ Absent	☐ Mild	☐ Moderate	☐ Severe
Dry appearance	☐ Absent	☐ Mild	☐ Moderate	☐ Severe
Edema	☐ Absent	☐ Mild	☐ Moderate	☐ Severe
If present:	☐ Acute	☐ Chronic		
If chronic:	☐ Pitting	☐ Nonpitting		
Ocular manifestations	☐ Absent	☐ Mild	☐ Moderate	☐ Severe
Peripheral location	☐ Absent	☐ Present		
If present:	List location(s)	______________________________________		
Phymatous changes	☐ Absent	☐ Mild	☐ Moderate	☐ Severe
Granulomatous changes	☐ Absent	☐ Mild	☐ Moderate	☐ Severe

Global Assessment

Physician ratings by subtype

Subtype 1: Erythematotelangiectatic	☐ Absent	☐ Mild	☐ Moderate	☐ Severe
Subtype 2: Papulopustular	☐ Absent	☐ Mild	☐ Moderate	☐ Severe
Subtype 3: Phymatous	☐ Absent	☐ Mild	☐ Moderate	☐ Severe
Subtype 4: Ocular	☐ Absent	☐ Mild	☐ Moderate	☐ Severe
Patient's global assessment	☐ Clear	☐ Mild	☐ Moderate	☐ Severe

Initial symptoms occurred: ____

Treatment prescribed: ____

Comments: ____

Physician: ____

Based on "Standard grading system for rosacea: Report of the National Rosacea Society Expert Committee on the Classification and Staging of Rosacea," *J Am Acad Dermatol* 2004;50:907-12.

PMA 362003

The Migraine Disability Assessment Test

The **MIDAS** (Migraine Disability Assessment) questionnaire was put together to help you measure the impact your headaches have on your life. The information on this questionnaire is also helpful for your primary care provider to determine the level of pain and disability caused by your headaches and to find the best treatment for you.

INSTRUCTIONS

Please answer the following questions about ALL of the headaches you have had over the last 3 months. Select your answer in the box next to each question. Select zero if you did not have the activity in the last 3 months. Please take the completed form to your healthcare professional.

_____ 1. On how many days in the last 3 months did you miss work or school because of your headaches?

_____ 2. How many days in the last 3 months was your productivity at work or school reduced by half or more because of your headaches? (Do not include days you counted in question 1 where you missed work or school.)

_____ 3. On how many days in the last 3 months did you not do household work (such as housework, home repairs and maintenance, shopping, caring for children and relatives) because of your headaches?

_____ 4. How many days in the last 3 months was your productivity in household work reduced by half of more because of your headaches? (Do not include days you counted in question 3 where you did not do household work.)

_____ 5. On how many days in the last 3 months did you miss family, social or leisure activities because of your headaches?

_____ Total (Questions 1-5)

What your Physician will need to know about your headache:

_____ A. On how many days in the last 3 months did you have a headache? (If a headache lasted more than 1 day, count each day.)

_____ B. On a scale of 0 - 10, on average how painful were these headaches? (where 0=no pain at all, and 10= pain as bad as it can be.)

Scoring: After you have filled out this questionnaire, add the total number of days from questions 1-5 (ignore A and B).

MIDAS Grade	Definition	MIDAS Score
I	Little or No Disability	0-5
II	Mild Disability	6-10
III	Moderate Disability	11-20
IV	Severe Disability	21+

If Your MIDAS Score is 6 or more, please discuss this with your doctor.

© Innovative Medical Research, 1997
© 2007, AstraZeneca Pharmaceuticals, LP. All Rights reserved.

References

1. Schaller M, Almeida LMC, Bewley A, et al. Recommendations for rosacea diagnosis, classification and management: update from the global ROSacea COnsensus 2019 panel. Br J Dermatol 2020; 182:1269-1276.
2. Wilkin J, Dahl M, Detmar M, et al. Standard grading system for rosacea: report of the National Rosacea Society Expert Committee on the classification and staging of rosacea. J Am Acad Dermatol 2004; 50:907-912.
3. Headache Classification Committee of the International Headache Society (IHS) The International Classification of Headache Disorders, 3rd edition. Cephalalgia 2018; 38:1-211.
4. Stewart WF, Lipton RB, Kolodner K, Liberman J, Sawyer J. Reliability of the migraine disability assessment score in a population-based sample of headache sufferers. Cephalalgia 1999; 19:107-114; discussion 174.
