# Supplementary material for: Evaluation of Dermatological and Neurological Aspects of the Relationship between Rosacea and Headaches
Source: Diagnostics (Basel). 2023 Dec 22;14(1):23. doi: 10.3390/diagnostics14010023 (PMC10795644; doi:10.3390/diagnostics14010023)
Supplement: Supplementary file 1 [file diagnostics-14-00023-s001.zip › Supplementary tables.pdf]

Supplementary Table S1. Demographic and clinical findings according to predominant subtypes of rosacea.

Variables	Predominant subtypes of rosacea			*P*-value
	ETR n=219	PPR n=68	RhR n=13	
Age, years	45.1 ± 12.3	45.8 ± 12.9	58.5 ± 4.5	0.001*
Gender, n (%)				
Male	55 (25.1)	18 (26.5)	13 (100.0)	<0.001*
Female	164 (74.9)	50 (73.5)	-	
BMI, kg/m^2	27.1 ± 4.5	27.3 ± 5.1	25.9 ± 3.4	0.619
Fitzpatrick skin type, n (%)				
Type II	52 (23.7)	23 (33.8)	3 (23.1)	0.257
Type III-IV	167 (76.3)	45 (66.2)	10 (76.9)	
Smoking, n (%)				
Never	160 (73.1)	58 (85.3)	5 (38.5)	
Current	46 (21.0)	4 (5.9)	5 (38.5)	0.001*
Former	13 (5.9)	6 (8.8)	3 (23.1)	
Alcohol use, n (%)	2 (0.9)	1 (1.5)	3 (23.1)	0.002*
History of family, n (%)	94 (42.9)	26 (38.2)	2 (15.4)	0.139
Comorbidities, n (%)	126 (57.5)	37 (54.4)	11 (84.6)	0.123
Peptic ulcer	119 (54.3)	34 (50.0)	9 (69.2)	0.440
Hypertension	52 (23.7)	17 (25.0)	7 (53.8)	0.068
Diabetes mellitus	37 (16.9)	14 (20.6)	2 (15.4)	0.775
Thyroid disease	20 (9.1)	11 (16.2)	1 (7.7)	0.206
Depression	11 (5.0)	3 (4.4)	2 (15.4)	0.288
Anxiety	6 (2.7)	1 (1.5)	-	0.999
Inflammatory bowel disease	2 (0.9)	1 (1.5)	-	0.814
Accompanying symptoms, n (%)				
Flushing	36 (16.6)	8 (11.8)	-	0.223
Burning, stinging, itching	81 (37.0)	28 (41.2)	2 (15.4)	0.217
Dryness	107 (48.9)	33 (48.5)	4 (30.8)	0.485
Facial edematous	14 (6.4)	5 (7.4)	4 (30.8)	0.020*
Granulamatous changes	1 (0.5)	-	-	0.999
Rosacea severity, n (%)				
Mild	81 (37.0)	14 (20.6)	-	
Moderate	93 (42.5)	35 (51.5)	9 (69.2)	0.004*
Severe	45 (20.5)	19 (27.9)	4 (30.8)	
Duration of disease, years	16 (2-50)	13.5 (1-40)	30 (10-40)	0.003*
Positive demodex test, n (%)	64 (29.2)	49 (72.1)	7 (53.8)	<0.001*
History of treatment, n (%)	39 (17.8)	32 (47.1)	-	<0.001*
Topical	35 (16.0)	30 (44.1)	-	<0.001*
Systemic	15 (6.8)	17 (25.0)	-	<0.001*
Triggering factors of rosacea, n (%)				
Sunlight	146 (66.7)	37 (54.4)	6 (46.2)	0.082
Stress	65 (29.7)	25 (36.8)	3 (23.1)	0.486
Heat	53 (24.2)	23 (33.8)	3 (23.1)	0.269
Spicy foods	42 (19.2)	22 (32.4)	2 (15.4)	0.065
Alcohol	1 (0.5)	-	1 (7.7)	0.131
Tea	11 (5.0)	3 (4.4)	-	0.999
Coffee	12 (5.5)	-	-	0.136
Menstruation	1 (0.5)	1 (1.5)	-	0.462
Cold	5 (2.3)	-	-	0.678

Data are mean ± standard deviation or median (min-max), or number (%). * p <0.05 shows statistical significance. BMI, body mass index; ETR, erythematotelangiectatic-type rosacea; PPR, papulopustular-type rosacea; RhR, phymatous-type rosacea.

Supplementary Table S2. Headache findings according to predominant subtypes of rosacea.

Variables	Predominant subtypes of rosacea			*P*-value
	ETR<br>n = 219	PPR<br>n = 68	RhR<br>n = 13	
Headache, n (%)	77 (35.2)	11 (16.2)	3 (23.1)	0.007*
TTH	17 (7.8)	6 (8.8)	3 (23.1)	0.004*
STH	9 (4.1)	1 (1.5)	-	
CTH	1 (0.5)	-	-	
Migraine	50 (22.8)	4 (5.9)	-	
Without episodic aura	31 (14.2)	2 (2.9)	-	0.023*
With episodic aura	18 (8.2)	2 (2.9)	-	
Menstrual migraine	1 (0.5)	-	-	
Headache onset, n (%)				
Before rosacea	15 (6.8)	2 (2.9)	-	0.046*
After rosacea	62 (28.2)	9 (13.2)	3 (23.1)	
Duration of headache onset, years	10 (1-25)	5 (1-15)	10 (1-30)	0.178
Headache severity, n (%)				
Mild	20 (9.1)	2 (2.9)	1 (7.7)	0.075
Moderate	27 (12.3)	5 (7.4)	2 (15.4)	
Severe	30 (13.7)	4 (5.9)	-	
MIDAS score	20 (3-60)	37 (12-49)	-	0.192
Migraine severity, n (%)				
Stage 1	3 (1.4)	-	-	0.137
Stage 2	14 (6.4)	1 (1.5)	-	
Stage 3	9 (4.1)	-	-	
Stage 4	24 (11.0)	3 (4.4)	-	
Triggering factors of headache, n (%)				
Stress	47 (21.5)	8 (11.8)	3 (23.1)	0.171
Coffee	7 (3.2)	-	-	0.416
Menstruation	14 (6.4)	2 (2.9)	-	0.531
Hypertension	7 (3.2)	-	-	0.416
Cheese	3 (1.4)	-	-	0.999

Data are mean ± standard deviation or median (min-max), or number (%). * *P*-value <0.05 shows statistical significance. CTH, cluster-type headache; ETR, erythematotelangiectatic-type rosacea; PPR, papulopustular-type rosacea; RhR, phymatous-type rosacea; STH, secondary-type headache; TTH, tension-type headache.

Supplementary Table S3. Demographic and clinical findings according to headache type in rosacea patients.

Variables	No headache n = 209	Type of Headache Migraine n = 54	TTH n = 26	STH n = 10	*P*-value
Age, years	47.2 ± 13.2	41.4 ± 9.4	42.4 ± 11	49.3 ± 10.9	0.007*
Female gender, n (%)	133 (63.6)	52 (96.3)	21 (80.8)	8 (80.0)	<0.001*
BMI, kg/m^2	27.2 ± 4.5	26.4 ± 4.9	26.6 ± 4.7	28.3 ± 4.2	0.484
Fitzpatrick skin type, n (%)					
Type II	56 (26.8)	14 (25.9)	8 (30.8)	-	0.258
Type III-IV	153 (73.2)	40 (74.1)	18 (69.2)	10 (100.0)	
Smoking, n (%)					
Never	160 (76.6)	37 (68.5)	17 (65.4)	9 (90.0)	
Current	34 (16.3)	12 (22.2)	7 (26.9)	1 (10.0)	0.664
Former	15 (7.2)	5 (9.3)	2 (7.7)	-	
Alcohol use, n (%)	4 (1.9)	1 (1.9)	1 (3.8)	-	0.703
History of family, n (%)	78 (37.3)	24 (44.4)	13 (50.0)	6 (60.0)	0.290
Comorbidities, n (%)	115 (55.0)	34 (63.0)	16 (61.5)	8 (80.0)	0.361
Peptic ulcer	104 (49.8)	34 (63.0)	16 (61.5)	7 (70.0)	0.195
Hypertension	57 (27.3)	9 (16.7)	6 (23.1)	4 (40.0)	0.277
Diabetes mellitus	34 (16.3)	9 (16.7)	6 (23.1)	4 (40.0)	0.222
Thyroid disease	23 (11.0)	6 (11.1)	-	3 (30.0)	0.084
Depression	14 (6.7)	-	1 (3.8)	1 (10.0)	0.116
Anxiety	6 (2.9)	-	-	1 (10.0)	0.232
Inflammatory bowel disease	3 (1.4)	-	-	-	0.999
Predominant subtypes of rosacea, n (%)					
ETR	142 (67.9)	50 (92.6)	17 (65.4)	9 (90.0)	
PPR	57 (27.3)	4 (7.4)	6 (23.1)	1 (10.0)	0.004*
PhR	10 (4.8)	-	3 (11.5)	-	
Accompanying symptoms, n (%)					
Flushing	30 (14.4)	6 (11.3)	6 (23.1)	2 (20.0)	0.466
Burning, stinging, itching	64 (30.6)	38 (70.4)	6 (23.1)	2 (20.0)	<0.001*
Dryness	93 (44.5)	34 (63.0)	10 (38.5)	6 (60.0)	0.065
Facial edematous	10 (18.5)	1 (3.8)	-	-	0.025*
Granulamatous changes		1 (1.9)	-	-	0.308
Rosacea severity, n (%)					
Mild	82 (39.2)	4 (7.4)	7 (26.9)	2 (20.0)	
Moderate	86 (41.1)	27 (50.0)	16 (61.5)	7 (70.0)	<0.001*
Severe	41 (19.6)	23 (42.6)	3 (11.5)	1 (10.0)	
Duration of disease, years	15 (1-50)	20 (4-47)	14.5 (3-40)	20 (5-30)	0.614
Positive demodex test, n (%)	87 (41.6)	22 (40.7)	8 (30.8)	3 (30.0)	0.777
History of treatment, n (%)	44 (21.1)	19 (35.2)	5 (19.2)	3 (30.0)	0.153
Topical	42 (20.1)	15 (27.8)	5 (19.2)	3 (30.0)	0.540
Systemic	22 (10.5)	8 (14.8)	1 (3.8)	1 (10.0)	0.507
Triggering factors of rosacea, n (%)					
Sunlight	122 (58.4)	41 (75.9)	19 (73.1)	6 (60.0)	0.074
Stress	58 (27.8)	25 (46.3)	8 (30.8)	2 (20.0)	0.068
Heat	57 (27.3)	16 (29.6)	2 (7.7)	4 (40.0)	0.081
Spicy foods	43 (20.6)	15 (27.8)	5 (19.2)	2 (20.0)	0.687
Alcohol	1 (0.5)	1 (1.9)	-	-	0.507
Tea	9 (4.3)	4 (7.4)	1 (3.8)	-	0.716
Coffee	6 (2.9)	5 (9.3)	1 (3.8)	-	0.173
Menstruation	1 (0.5)	1 (1.9)	-	-	0.507
Cold	2 (1.0)	1 (1.9)	1 (3.8)	1 (10.0)	0.064

Data are mean ± standard deviation or median (min-max), or number (%). *P*-value <0.05 shows statistical significance. BMI, body mass index; ETR, erythematotelangiectatic-type rosacea; PPR, papulopustular-type rosacea; RhR, phymatous-type rosacea; STH, secondary-type headache; TTH, tension-type headache

Supplementary Table S4. Other findings according to headache type in rosacea patients.

Variables	Type of Headache			P-value
	Migraine<br>n = 54	TTH<br>n = 26	STH<br>n = 10	
Headache onset, n (%)				
Before rosacea	10 (18.5)	5 (19.2)	1 (10.0)	0.925
After rosacea	44 (81.5)	21 (80.8)	9 (90.0)	
Duration of headache onset, years	8 (1-25)	10 (1-30)	15 (3-20)	0.196
Headache severity, n (%)				
Mild	9 (16.7)	8 (30.8)	6 (60.0)	<0.001*
Moderate	15 (27.8)	16 (61.5)	3 (30.0)	
Severe	30 (55.6)	2 (7.7)	1 (10.0)	
Triggering factors of headache, n (%)				
Stress	34 (63.0)	24 (92.3)	-	<0.001*
Coffee	7 (13.0)	-	-	<0.001*
Menstruation	16 (29.6)	-	-	<0.001*
Hypertension	-	1 (3.8)	6 (60.0)	<0.001*
Cheese	3 (5.6)	-	-	0.022*

Data are mean ± standard deviation or median (min-max), or number (%). *P*-value <0.05 shows statistical significance. STH, secondary-type headache; TTH, tension-type headache

Supplementary Table S5. Common trigger factors for headache and rosacea patients.

	Triggering factors	Rosacea								
		Sunlight n=189	Stress n=93	Heat n=79	Spicy foods n=66	Alcohol n=2	Tea n=14	Coffee n=12	Menstruation n=2	Cold n=5
Headache	Sunlight	-	-	-	-	-	-	-	-	-
	Stress	47 (24.9)	**28 (30.1)**	11 (13.9)	14 (21.2)	1 (50.0)	4 (28.6)	7 (41.7)	-	1 (20.0)
	Heat	-	-	-	-	-	-	-	-	-
	Spicy foods	-	-	-	-	-	-	-	-	-
	Alcohol	-	-	-	-	**1 (50.0)**	-	-	-	-
	Tea	-	-	-	-	-	-	-	-	-
	Coffee	5 (2.6)	4 (4.3)	1 (1.3)	-	-	-	**2 (16.7)**	1 (50.0)	-
	Menstruation	13 (6.9)	3 (3.2)	7 (7.6)	7 (10.6)	-	3 (21.4)	1 (8.3)	**1 (50.0)**	-
	Cold	-	-	-	-	-	-	-	-	-
	Hypertension	3 (1.6)	1 (1.1)	2 (2.5)	1 (1.5)	-	-	-	-	1 (20.0)
	Cheese	3 (1.6)	1 (1.1)	1 (1.3)	1 (1.5)	-	-	-	-	-

Bold characters indicate common triggering factors.
